# Diabetes and respiratory diseases as comorbid conditions in COVID-19 patients from Kazakhstan: a retrospective pilot study

**DOI:** 10.7717/peerj.20413

**Published:** 2025-12-16

**Authors:** Karlygash Tazhibayeva, Zinat Abdrakhmanova, Ariel Shensa, Aizhan Raushanova, Assel Sadykova, Natalya Glushkova, Saltanat Mamyrbekova, Zhanar Zhagiparova, Arailym Beisbekova, Faina Linkov

**Affiliations:** 1 Al-Farabi Kazakh National University, Almaty, Kazakhstan; 2 Duquesne University, Pittsburgh, PA, United States of America; 3 Kazakh National Medical University Named After S. D. Asfendiyarov, Almaty, Kazakhstan

**Keywords:** Central Asia, Disease burden, Diabetes, Kazakhstan, Prevention

## Abstract

**Background:**

Diabetes and respiratory diseases are prevalent in Central Asia, and their prevention represents an opportunity to increase life expectancy in the region, especially in Kazakhstan. This study aims to analyze trends in the prevalence of diabetes and respiratory diseases as comorbidities in patients with recent COVID-19 infections, based on the information obtained from several clinics in Almaty, Kazakhstan.

**Methods:**

This study was conducted as a cross-sectional analysis using de-identified electronic medical records from a multidisciplinary hospital in Almaty, Kazakhstan. Adults hospitalized with laboratory-confirmed COVID-19 during 2021–2022 were included (*N* = 292; mean age 55 years, range 18–91). Continuous variables were summarized with means and standard deviations, and categorical variables with counts and percentages. Data extraction and statistical analyses were performed in September–December 2023.

**Results:**

The mortality rate was higher among patients with pneumonia (16.8%) compared to chronic bronchitis (10.6%). We identified a high level of comorbidity with diabetes (35.3% in the 45–59 age group) and chronic respiratory disease (37.9% in the same group).

**Discussion:**

The mortality and comorbidity rates for hospitalized patients with recent COVID-19 infections in Almaty were high in comparison to other middle-income countries. These findings underscore the need for targeted early prevention strategies and public health interventions to address the rising burden of chronic diseases in Kazakhstan, particularly among middle-aged adults.

## Introduction

The recent COVID-19 pandemic raised significant concerns about health care outcomes of individuals diagnosed with COVID-19 infection experiencing comorbid conditions, particularly respiratory diseases, such as chronic bronchitis and pneumonia (Pya et al., 2021). In 2017, an estimated 544.9 million people worldwide were living with chronic respiratory diseases (CRDs)—a 39.8% increase since 1990 (Soriano et al., 2020). Using Global Burden of Disease Study (GBD) 2019 estimates, CRDs ranked as the third-leading cause of death globally; however, according to WHO Global Health Estimates for 2021, ischemic heart disease was the leading cause, COVID-19 ranked second, stroke third, and chronic obstructive pulmonary disease fourth (Momtazmanesh et al., 2023; Otegenova et al., 2024).

Chronic bronchitis, one of the most common respiratory diseases, is of particular concern due to its association with severe respiratory complications, especially in the context of COVID-19. COVID-19 frequently leads to pneumonia, significantly worsening outcomes for patients with pre-existing respiratory diseases, such as chronic bronchitis. These patients often require hospitalization, intensive care, and long-term management of complications, such as interstitial lung diseases and pulmonary vascular disease (Gülsen et al., 2021; Iskakova et al., 2023).

Low and middle-income countries bear a heavy burden of respiratory diseases and multimorbidity, placing a tremendous strain on healthcare systems (Abebe et al., 2020; George et al., 2020). The global burden of chronic diseases, particularly respiratory diseases, has been steadily increasing over the past few decades. These diseases not only lead to significant morbidity and mortality but also lead to increasing healthcare systems burden worldwide (Patterson et al., 2009). It is predicted that by 2035, the number of people suffering from diabetes could rise to 592 million, particularly in low and middle-income countries (Divers et al., 2020). The presence of both diabetes and respiratory diseases in a single patient significantly increases the risk of severe COVID-19 progression and mortality (Liu et al., 2020). These comorbid conditions exacerbate adverse health events, leading to a deterioration in the patient’s overall condition (Gold et al., 2020).

Global data indicated a decline in the mortality rate from chronic respiratory diseases by 2.41% annually between 1990 and 2017. However, regions with low social-demographic indices continue to exhibit high mortality rates from respiratory diseases, suggesting the need to address preventable causes of mortality in countries like Kazakhstan (Kosherbayeva et al., 2024).

Kazakhstan, like many other countries, faces challenges associated with an aging population, which has led to an increase in the prevalence of chronic diseases, including chronic bronchitis. Smoking, air pollution, and occupational exposures are significant risk factors contributing to the high prevalence of respiratory diseases in Kazakhstan. While the mortality rate from lower respiratory infections has been declining since 1991, there has been a notable rise in respiratory-related deaths among older age groups, exacerbated by limited healthcare resources (Supiyev, 2018; Otegenova et al., 2024). Due to the potential underreporting of COVID-19 cases, conducting studies on the interrelationship between COVID-19 and other respiratory infections is challenging (Rezaei, 2024).

The prevalence of chronic bronchitis and its co-occurrence with COVID-19 in Kazakhstan remains underreported, though early data suggest these comorbidities are linked to higher mortality rates and more severe clinical outcomes (Chen et al., 2023). Unlike asthma, which has shown no consistent association with severe COVID-19 progression, chronic bronchitis poses a significant risk, emphasizing the need for focused prevention and treatment strategies (Dyusupova et al., 2021).

Patients with respiratory diseases, particularly chronic bronchitis, should be classified as a high-risk group and receive appropriate treatment and monitoring. This includes regular lung function assessments, early interventions to address symptom exacerbations, and robust infection prevention measures (Sanyaolu et al., 2020; Marron et al., 2021). Ensuring access to medical resources for treating COVID-19 and its complications is equally critical (Cheng et al., 2021). Special attention should be given to infection prevention in patients with chronic respiratory diseases, as well as ensuring access to essential medical resources for the treatment of COVID-19 and its complications (Mahumud, Kamara & Renzaho, 2020).

The increasing burden of diabetes and chronic respiratory diseases among COVID-19 patients is not unique to Kazakhstan but reflects a broader global trend, particularly in low- and middle-income countries (LMICs). Understanding the interplay of these comorbidities is critical for developing targeted public health interventions applicable both regionally and internationally.

Thus, the aim of this study is to analyze trends in the prevalence of diabetes and respiratory diseases as comorbidities in patients presenting with recent COVID-19 infections, based on the information obtained from several clinics in Almaty, Kazakhstan. We aimed to look at the burden of these conditions by age groups using medical record review approaches, testing of biological samples, and evaluating data using existing medical records. Our ultimate aim is to provide policy recommendations for the reduction of comorbidity burden and improving primary prevention activities in Kazakhstan and Central Asia.

## Materials & Methods

### Study design

This cross-sectional study is aimed at identifying contributing factors to adverse outcomes associated with COVID-19 infection among patients hospitalized for COVID-19. This study was based electronic medical records (EMRs) of patients hospitalized with laboratory-confirmed COVID-19 in Almaty, Kazakhstan. The EMR data reflected acute COVID-19 cases admitted between March 2021 and December 2022, covering several epidemic waves in the region. Data extraction, cleaning, and statistical analysis were conducted between September and December 2023. This clarification ensures that the study design is consistent with the retrospective nature of the dataset.

### Sampling and participants

We recruited a sample of 292 research participants from a total of 524 potentially eligible patients monitored in clinics and regional hospitals in Almaty. The sample size was determined using Epi Info, a statistical software developed by the Centers or Disease Control and Prevention (CDC) (Division of Health Informatics & Surveillance (DHIS), Center for Surveillance, Epidemiology & Laboratory Services (CSELS), 2020). We retrospectively reviewed electronic medical records (EMRs) of patients hospitalized with laboratory-confirmed COVID-19 in Almaty, Kazakhstan. The catchment population of the participating institutions was approximately 150,000 residents at the time of the study. From EMRs, 524 records were identified as potentially eligible. After screening, 232 cases were excluded due to incomplete records (*n* = 124), not meeting inclusion criteria or having unrelated comorbidities (*n* = 68), or missing key outcome variables (*n* = 40). The final sample comprised 292 patients.

**Figure 1:**
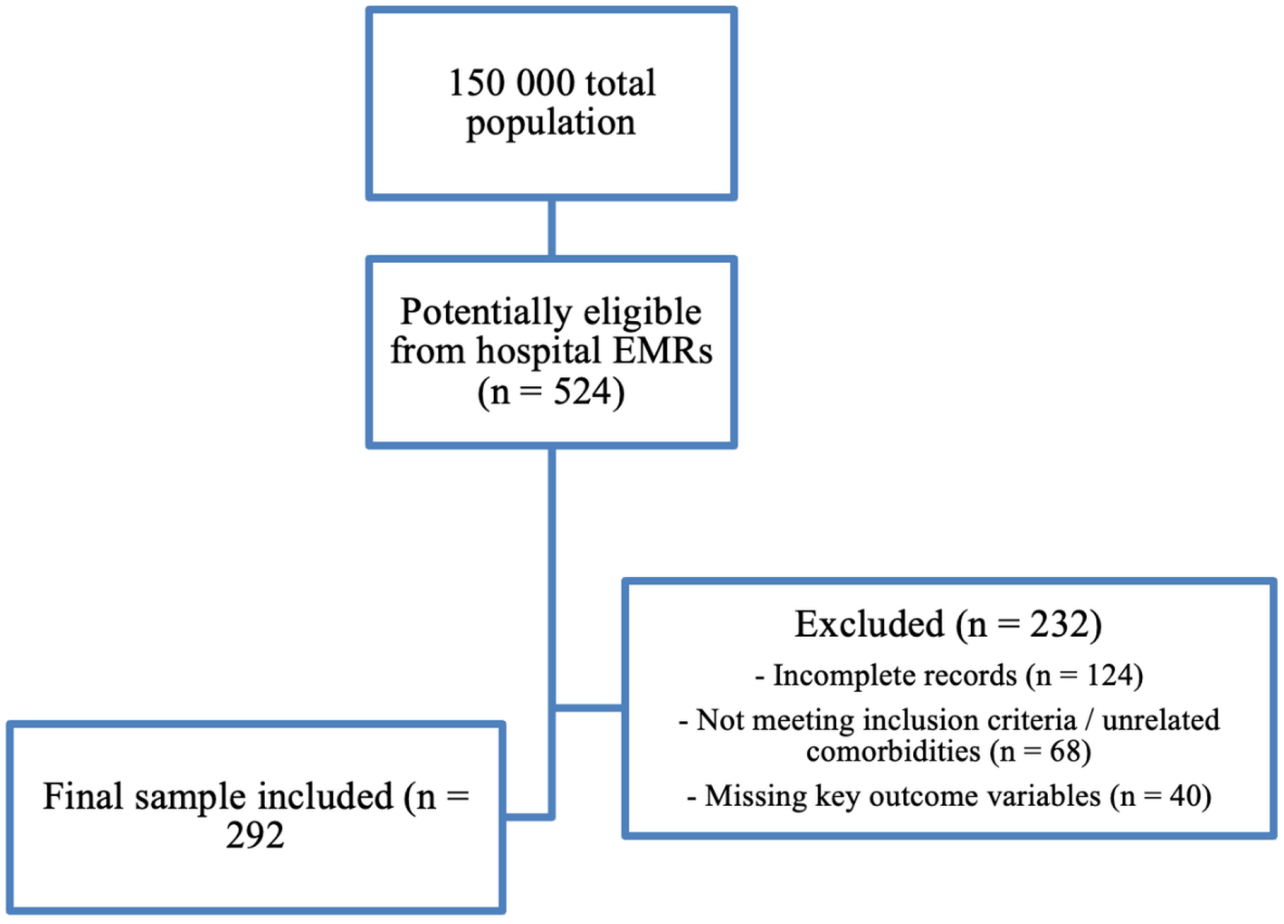
Participant sampling flow diagram for COVID-19 EMR cohort in Kazakhstan. From a source population of ∼150,000, 524 records were screened from hospital EMRs; 232 were excluded (incomplete records, unrelated comorbidities/not meeting inclusion criteria, or missing key outcome variables), yielding a final analytic sample of 292 patients.

All included patients had at least one documented comorbid condition (diabetes mellitus, pneumonia, or chronic bronchitis). Consequently, our comparisons are performed across comorbidity subgroups rather than against a non-comorbid control group. The flow of participants is presented in Fig. 1.

### Ethics statement

Ethical approval for this study was granted by the Local Ethics Committee (LEC) of Al-Farabi Kazakh National University on August 17, 2023 (Approval No. 74). The study utilized a cross-sectional design (retrospective) based on fully anonymized patient records, and the LEC waived the requirement for informed consent due to the de - identified nature of the data.

### Data dollection and statistical analysis

Data were collected from the following sources: electronic medical records (EMRs) which included documented comorbidities, such as diabetes, pneumonia, and chronic bronchitis based on medical history and physician diagnoses. Laboratory methods: nasopharyngeal swabs were collected for polymerase chain reaction (PCR) testing to confirm COVID-19 infection within 24 h of symptom presentation.

Descriptive statistics were used to calculate means and standard deviations for continuous variables, such as age, while categorical variables, including gender and the presence of comorbid conditions (diabetes, chronic bronchitis, and pneumonia), were described using frequencies and percentages. Chi-square tests were conducted to assess the association between categorical variables, such as gender and the presence of specific comorbid conditions (diabetes, chronic bronchitis, and pneumonia) in patients with a confirmed COVID-19 diagnosis. Additionally, Chi-square tests were used to examine the associations between age groups and the prevalence of these comorbid conditions. Correlation analysis was performed to evaluate the strength and direction of the relationship between continuous variables, such as age, and the prevalence of chronic diseases like diabetes and respiratory conditions. Gender was also analyzed as a categorical variable to determine its correlation with the distribution of comorbidities.

Multivariable logistic regression models were employed to evaluate the independent effects of age, gender, and comorbid conditions (diabetes, pneumonia, and chronic bronchitis) on four binary clinical outcomes: (1) mortality (death *vs.* survival), (2) clinical improvement (defined as discharge with resolution of symptoms and no readmission within 14 days), (3) no change (defined as unchanged condition or prolonged hospitalization without deterioration), and (4) need for mechanical ventilation (yes/no). Each outcome was analyzed using a separate multivariable logistic model, with adjusted odds ratios (AOR) and 95% confidence intervals (CI) reported.

Model assumptions were verified, including multicollinearity (variance inflation factor (VIF) < 10) and linearity of log odds (using Box-Tidwell tests). Correction for multiple testing was performed using the Benjamini–Hochberg false discovery rate (FDR) method. Diabetes was included as an independent covariate in all regression models, to directly address the study’s central hypothesis.

## Results

The study included 292 patients with a mean age of 55 years (range 18–91 years), of whom 104 (35.7%) were male. The study sample predominantly consisted of younger to middle-aged adults, with the majority falling into the 18–44 years age group (41.1%), followed by those aged 45–60 years (31.5%), and the remaining >60 years group representing 27.4% of the total. Among the total population with confirmed COVID-19, the most common comorbid conditions were pneumonia and chronic bronchitis. Pneumonia was present in 166 (56.8%) of the participants, while chronic bronchitis affected 126 (43.2%). Pneumonia had the highest prevalence in the 45–60 years age group, accounting for 46.7% of participants with this condition. Chronic bronchitis was most prevalent in the same age group, with 42.1% of participants affected, followed by 24.6% in the 18–44 years age group and 29.4% in those aged over 60 years. Table 1 outlines basic clinical characteristics of the study participants. It illustrates the demographic and clinical outcomes, including mortality (Death), clinical improvement (Improvement), change in condition (No Change), and the need for artificial ventilation (Artificial Ventilation). Specifically, 27% of patients died in the hospital due to complications. For each outcome, the number of patients (N), percentage (%), and *p*-values are presented, highlighting the statistical significance of each observation.

**Table 1:** Total study sample demographic and clinical characteristics and bivariable associations with clinical outcomes. Adults hospitalized with laboratory-confirmed COVID-19 in Almaty, Kazakhstan (*N* = 292). Rows report counts and row percentages for age groups (18–44, 45–60, >60), sex, and comorbidities (pneumonia, chronic bronchial disease) across four outcomes (death, improvement, no change, artificial ventilation). *P*-values are from Pearson’s *χ*^2^ (Yates for 2 × 2; Fisher exact when expected counts <5). Reference categories: age 18–44 years, female sex, and absence of comorbidity

Characteristic	Total study sample N (%)	Clinical outcomes				*P* value^*^
		Death n (%)	Improvement n (%)	No change n (%)	Artificial ventilation n (%)	
Age						0.042
18–44 years	117 (41.1)	28 (23.9)	65 (55.6)	6 (5.1)	18 (15.4)	
45–60 years	95 (31.5)	23 (24.2)	42 (44.2)	3 (3.2)	27 (28.4)	
>60 years	80 (27.4)	28 (35)	11 (13.8)	6 (7.4)	35 (43.8)	
Gender						0.340
Female	188 (64.3)	54 (28.7)	72 (38.3)	9 (4.8)	53 (28.2)	
Male	104 (35.7)	28 (26.9)	40 (38.5)	6 (5.8)	30 (28.8)	
Comorbidity						
Pneumonia	166 (56.8)	49 (29.5)	59 (35.5)	11 (6.6)	47 (28.3)	0.424
Chronic bronchitis	126 (43.2)	31 (24.6)	53 (42.1)	5 (3.9)	37 (29.4)	0.311

**Notes.**

* Age categories defined as follows: 18–44 years (≤44 years), 45–60 years (45–60 years inclusive), and >60 years (≥61 years). Percentages are calculated within each subgroup (row percentages). Reference categories: age 18–44 years, female sex, and absence of comorbidity.

Table 2 provides insights into the adjusted odds ratios (AORs) and 95% confidence intervals (CIs) for clinical outcomes in COVID-19 patients based on demographic characteristics (age and gender) and comorbidities (pneumonia and chronic bronchitis). The outcomes analyzed include Death, Improvement, No Change, and the Need for Artificial Ventilation.

**Table 2:** Adjusted odds ratios (AOR) and 95% confidence intervals (CI) for demographic and clinical predictors of COVID-19 outcomes (*N* = 292). Multivariable (multinomial) logistic regression estimating AORs (95% CI) for age group, sex, pneumonia, chronic bronchial disease, and diabetes; models adjusted for all listed covariates with *p*-values FDR-corrected (Benjamini–Hochberg). Reference categories: age 18–44 years, female sex, no pneumonia, no chronic bronchial disease, no diabetes. AORs are presented separately for four clinical outcomes (death, improvement, no change, mechanical ventilation); model assumptions checked (VIF, Box–Tidwell), details in Methods.

**Predictor**	Clinical outcomes			
	Death AOR (95% CI)	Improvement AOR (95% CI)	No Change AOR (95% CI)	Mechanical ventilation AOR (95% CI)
18–44 years	1.0 (ref)	1.0 (ref)	1.0 (ref)	1.0 (ref)
Age 45–60	1.6 (1.2–2.1)	0.7 (0.5–1.0)	1.1 (0.8–1.5)	1.8 (1.4–2.4)
Age > 60	2.4 (1.8–3.2)	0.4 (0.3–0.7)	1.6 (1.2–2.2)	3.0 (2.2–4.0)
Male (*vs*. Female)	1.5 (1.2–1.9)	0.8 (0.6–1.0)	1.4 (1.1–1.8)	1.6 (1.2–2.1)
Pneumonia	1.8 (1.4–2.3)	0.6 (0.4–0.8)	1.7 (1.3–2.2)	2.2 (1.7–2.9)
Chronic bronchitis	1.4 (1.1–1.8)	0.9 (0.7–1.2)	1.5 (1.2–2.0)	1.8 (1.4–2.3)
Diabetes	1.7 (1.3–2.2)	0.7 (0.5–0.9)	1.4 (1.1–1.8)	2.1 (1.6–2.7)

**Notes.**

AOR, adjusted odds ratio; CI, confidence interval. All models adjusted for age, gender, pneumonia, chronic bronchitis, and diabetes. *P*-values corrected using FDR.

Reference categories: age 18–44 years, female sex, no pneumonia, no chronic bronchitis, no diabetes.

Older patients were at a much higher risk of experiencing severe outcomes. Those aged 45–60 years had 1.6 times higher odds of dying compared to younger adults (18–44 years), while patients over 60 years had more than double the risk (2.4 times higher). On the other hand, the odds of improvement decreased significantly with age, and patients over 60 had the lowest odds of recovery. The need for artificial ventilation was especially high in the oldest age group, with the odds being three times greater than for younger adults. Male patients were at greater risk of adverse outcomes compared to females. They were 1.5 times more likely to die and 1.6 times more likely to require artificial ventilation. However, their odds of improvement were slightly lower than for females. Pneumonia had a strong negative impact on outcomes. Patients with pneumonia were 1.8 times more likely to die and 2.2 times more likely to need artificial ventilation compared to those without pneumonia. Their chances of improvement were also significantly lower. Chronic bronchitis also posed risks but to a lesser extent than pneumonia. It increased the odds of death and the need for ventilation, although its impact on improvement was less severe. This table highlights the critical role that age, gender, and comorbidities play in determining outcomes for COVID-19 patients.

Diabetes was independently associated with all adverse COVID-19 outcomes. Patients with diabetes had 1.7 times higher odds of death (95% CI [1.3–2.2]) and more than twice the odds of requiring mechanical ventilation (AOR: 2.1, 95% CI [1.6–2.7]). Their likelihood of clinical improvement was significantly lower (AOR: 0.7, 95% CI [0.5–0.9]), and they were more likely to experience no change in condition (AOR: 1.4, 95% CI [1.1–1.8]). These results underscore the central role of diabetes in worsening COVID-19 prognosis, independent of other comorbidities.

Table 3 compares the clinical outcomes of COVID-19 patients with diabetes who also have either chronic bronchitis or pneumonia as a comorbidity. The outcomes investigated include Death, Improvement, No Change, and Transition to Artificial Ventilation, expressed as percentages, along with odds ratios (OR) and 95% confidence intervals.

**Table 3:** Bivariable associations between Covid-19 comorbidities and clinical outcomes. Unadjusted odds ratios (OR, 95% CI) comparing presence *vs* absence of each comorbidity (pneumonia, chronic bronchial disease) across four clinical outcomes (death, improvement, no change, mechanical ventilation) in the total study sample (*N* = 292).

Comorbidity^*^	Total study sample (*N* = 292) %	Clinical outcomes			
		Death	Improvement	No change	Artificial ventilation
OR (95% CI)^**^					
Pneumonia					
Yes	56.8	1.8 (1.4–2.3)	0.6 (0.4–0.8)	1.7 (1.3–2.2)	2.2 (1.7–2.9)
No	43.2	1.0 (ref)	1.0 (ref)	1.0 (ref)	1.0 (ref)
Chronic bronchitis					
Yes	43.2	1.4 (1.1–1.8)	0.9 (0.7–1.2)	1.5 (1.2–2.0)	1.8 (1.4–2.3)
No	56.8	1.0 (ref)	1.0 (ref)	1.0 (ref)	1.0 (ref)

**Notes.**

* Comorbidities occurring in patients with COVID-19 and diabetes. Reference category: absence of comorbidity. Odds ratios derived from Chi-square tests

** Derived from Chi-square tests.

Chi-square tests revealed statistically significant associations between comorbidities and clinical outcomes.

Patients with chronic bronchitis exhibited elevated risks across several outcomes. The odds of death were significantly higher (OR: 1.4, 95% CI [1.1–1.8]), while the likelihood of improvement was slightly reduced (OR: 0.9, 95% CI [0.7–1.2]). Additionally, the odds of experiencing no change in clinical condition were increased (OR: 1.5, 95% CI [1.2–2.0]), and the need for artificial ventilation was significantly higher (OR: 1.8, 95% CI [1.4–2.3]).

Patients with pneumonia faced even worse outcomes, with significantly higher odds of death (OR: 1.8, 95% CI [1.4–2.3]) and artificial ventilation (OR: 2.2, 95% CI [1.7–2.9]). Improvement rates were substantially lower in pneumonia patients (OR: 0.6, 95% CI [0.4–0.8]), while the odds of no change were also elevated (OR: 1.7, 95% CI [1.3–2.2]). These findings were supported by chi-square tests, revealing statistically significant associations across all clinical outcomes (*p* < 0.05).

Mortality was notably higher among patients with pneumonia (16.8%) compared to those with chronic bronchitis (10.6%), with a *p*-value of 0.005. Improvement rates were greater in the chronic bronchitis group (18.2%) than in the pneumonia group (9.9%), *p* = 0.03. Similarly, the need for artificial ventilation was more frequent in pneumonia cases (19.9%) than in chronic bronchitis cases (11.3%), *p* = 0.001.

These findings highlight the heightened risks associated with chronic bronchitis and pneumonia in COVID-19 patients with diabetes, emphasizing the need for targeted interventions and close monitoring of these high-risk groups.

## Discussion

Our key finding is that higher mortality and ventilation rates for COVID-19 patients were observed in those with confirmed pneumonia. We also observed very high COVID-19 mortality rate among pneumonia patients in comparison to other middle income countries (Khedr et al., 2022), with studies indicating that the prevalence of severe respiratory complications and secondary bacterial infections significantly contributes to this outcome. As this group of patients exhibited an alarmingly high mortality rate, particularly among those with severe comorbid conditions, there is a critical need to explore the critical impact of comorbidities on COVID-19 outcomes.

Our revised analysis confirms that diabetes is a significant independent risk factor for adverse COVID-19 outcomes in hospitalized patients. Patients with diabetes had substantially higher odds of death and the need for mechanical ventilation, even after controlling for age, gender, and other respiratory comorbidities. These findings are consistent with global studies showing that hyperglycemia and insulin resistance contribute to immune dysregulation, delayed viral clearance, and increased inflammatory response in COVID-19 patients (Cheng et al., 2021; Chen et al., 2023; Gazezova et al., 2023).

The inclusion of diabetes in all multivariable models strengthens the coherence between our research question and statistical approach. In contrast to chronic bronchitis, which showed a moderate association with poor outcomes, diabetes had a more consistent and pronounced impact across all outcome domains. Pneumonia remained the strongest respiratory predictor of death, but diabetes emerged as a parallel driver of severity, especially among middle-aged and older adults. This dual burden reflects the reality in Kazakhstan, where the prevalence of type 2 diabetes is rising and often coexists with underdiagnosed respiratory conditions (Soriano et al., 2020; Kosherbayeva et al., 2024).

The combination of diabetes and pneumonia significantly amplified clinical risk, suggesting synergistic interactions between metabolic and respiratory pathways. These data support targeted early intervention and multidisciplinary management strategies in COVID-19 patients with chronic conditions. Importantly, our findings also highlight the need for improved glycemic control protocols in inpatient COVID-19 care, especially in middle-income countries facing dual challenges of noncommunicable and infectious disease burdens.

Previously published research from Kazakhstan highlighted that pneumonia remains a leading cause of hospitalization and death, particularly in older adults with comorbid conditions such as diabetes and chronic respiratory diseases. According to Zhussupov et al. (2021) pneumonia cases during the COVID-19 pandemic in Kazakhstan were characterized by delayed hospital admissions, limited access to critical care resources, and high prevalence of multidrug-resistant bacterial infections, all of which compounded mortality risks (Smagul et al., 2022). The increased mortality in pneumonia cases can also be attributed to Kazakhstan’s environmental and healthcare challenges. Studies by Kerimray et al. (2020) emphasize that high levels of air pollution, including particulate matter in urban centers like Almaty, contribute to a higher baseline prevalence of chronic respiratory diseases, which exacerbate the progression of pneumonia in COVID-19 patients. Furthermore, smoking prevalence, reported to be over 25% among adults in Kazakhstan, has been strongly linked to both chronic bronchitis and pneumonia, compounding the severity of respiratory infections (Kazakhstan, Office for Prevention & Control of NCDs (MOS), Tobacco (TOB), WHO Europe, 2019).

Our findings, coupled with published studies, suggest that pneumonia substantially increases the risk of severe respiratory failure requiring mechanical support. In this study, pneumonia is linked to higher mortality, a greater need for mechanical ventilation, and less improvement compared to chronic bronchitis. These statistically significant differences emphasize the importance of tailored clinical strategies based on whether patients have chronic bronchitis or pneumonia as a comorbidity with COVID-19 and diabetes.

The findings of this study also provide valuable insights into the clinical outcomes of COVID-19 patients with diabetes. The most substantial impact of these conditions was observed in the 45–60 years age group, indicating that middle-aged adults with these comorbidities may experience more severe clinical outcomes. It reinforces the need to focus care and resources on older adults, male patients, and those with pneumonia or chronic bronchitis to improve their chances of recovery.

This trend is consistent with other studies conducted in Kazakhstan that highlight the high prevalence of COVID-19 and associated complications among older male populations (Yegorov et al., 2021; Zhussupov et al., 2021). Previous research in Central Asia has also shown that older adults, especially males, have higher rates of diabetes and respiratory illnesses, leading to more severe COVID-19 outcomes (Almagro et al., 2020; Steinthorsdottir et al., 2020). This pattern emphasizes the need for targeted healthcare strategies to manage these comorbidities effectively, particularly among middle-aged COVID-19 patients.

The prevalence of pneumonia and chronic bronchitis observed in this study reflects the broader disease trends within Kazakhstan. Chronic respiratory diseases are widespread, partly due to high smoking rates and environmental factors such as air pollution, which are especially prominent in industrialized regions like Almaty (Kerimray et al., 2020). These factors contribute to the higher rates of chronic bronchitis found among middle-aged and older adults.

Our analysis shows a statistically significant difference in clinical outcomes between patients with chronic bronchitis and those with pneumonia. Specifically, among COVID-19 patients with diabetes, those with pneumonia had higher mortality rates and a greater need for artificial ventilation compared to those with chronic bronchitis, indicating that pneumonia poses a greater risk in this patient population. This finding aligns with global studies indicating that pneumonia, particularly bacterial pneumonia as a secondary infection, significantly worsens COVID-19 prognosis (WHO, 2021; Scott et al., 2022). Kazakhstan has reported challenges in managing COVID-19 complications, especially in patients with comorbidities such as diabetes (Gazezova et al., 2023). The healthcare system in Kazakhstan, while rapidly developing, faces resource limitations that affect the management of severe respiratory conditions. This is evident in the higher need for artificial ventilation in pneumonia patients, suggesting that pneumonia presents a critical challenge that requires targeted healthcare strategies, such as early intervention protocols and enhanced monitoring in diabetic patients.

The improvement rate among chronic bronchitis patients (18.2%) compared to pneumonia patients (9.9%) suggests that chronic bronchitis, despite being a long-term condition, may allow for better management outcomes when compared to the acute onset of pneumonia. This finding is in line with studies from other regions showing that chronic bronchitis patients, when managed with bronchodilators and steroids, can achieve good outcomes even when infected with COVID-19 (Bai et al., 2022). However, the higher mortality and ventilation rates among pneumonia patients reflect Kazakhstan’s broader need to enhance its critical care capacity, particularly for respiratory diseases. Pneumonia remains a leading cause of hospital admissions and mortality in the country, highlighting the urgency for improved diagnostic capabilities and access to ventilators (Vinnikov et al., 2023).

Our findings align with global research showing that diabetes and chronic respiratory conditions significantly worsen COVID-19 outcomes. Similar trends have been observed in other middle-income countries, including Brazil, India, and South Africa, where high rates of air pollution and smoking exacerbate respiratory disease prevalence. These results suggest that public health interventions targeting multimorbidity in COVID-19 patients should be prioritized not only in Kazakhstan but also in other LMICs facing similar epidemiological challenges.

### Additional limitations

Several additional limitations should be noted. First, although our dataset was retrospective, data extraction and analysis occurred in September–December 2023, while the EMR cases themselves reflected acute hospitalizations from earlier pandemic waves (2021–2022). This distinction clarifies the study period. A major limitation of our study is that we did not include a control group of COVID-19 patients without comorbidities. All participants had at least one documented condition (diabetes, pneumonia, or chronic bronchitis). Therefore, our analysis compares outcomes across different comorbidity subgroups, but it does not allow us to evaluate the relative risk compared to patients without comorbidities. This limitation should be taken into account when interpreting the findings, and future studies should incorporate non-comorbid COVID-19 cohorts to better quantify relative risks.

Second, potential confounding factors such as smoking status, vaccination status, prior COVID-19 infection, and body mass index (BMI) were not available in the EMR dataset. Each of these factors has been shown in previous research to significantly influence COVID-19 outcomes: smoking increases severity of respiratory illness (George et al., 2020), vaccination reduces hospitalization and mortality (Kozhekenova et al., 2025), reinfections can follow a different clinical trajectory than primary cases (Kassabekova et al., 2025), and obesity is a strong predictor of adverse prognosis (Costanzo et al., 2015). Their absence should be considered when interpreting our findings. In our dataset, vaccination status could not be included because, during 2021–2022 in Kazakhstan, vaccination information was captured in the national registry and issued *via* eGov Mobile digital certificates rather than in hospital EMRs, precluding record linkage; this limitation should be considered when interpreting our findings.

Third, our dataset did not consistently capture additional comorbidities such as hypertension, cardiovascular disease, dyslipidemia, chronic obstructive pulmonary disease (COPD), asthma, or immunosuppression. These conditions are known to interact with diabetes and respiratory illness to worsen prognosis (Smagulov et al., 2025). Future research in Kazakhstan should expand EMR systems to capture multimorbidity in greater detail.

Fourth, the lack of vaccination data is a critical limitation. Global studies confirm that COVID-19 vaccination reduces severity, intensive care unit (ICU) admission, and death (Kassabekova et al., 2025). Even though this information was not available in our EMRs, we emphasize that vaccination likely influenced outcomes in our population.

Finally, variations in clinical management and treatment should be acknowledged. During the study period, Kazakhstani hospitals primarily used corticosteroids and anticoagulants for COVID-19 management, with limited access to antiviral drugs such as remdesivir, molnupiravir, or nirmatrelvir/ritonavir (WHO, 2021). These limitations in treatment options likely contributed to the higher mortality observed in pneumonia patients.

The strengths of this study include our ability to look at the population that is rarely addressed by the current research literature (Kazakhstani residents with multimorbidity) and robust methodology. The weaknesses of this study include our relatively small sample size and potential inability to generalize to rural settings.

### Conclusion

In conclusion, our findings demonstrate that diabetes is a major predictor of mortality, clinical deterioration, and the need for mechanical ventilation among COVID-19 patients in Kazakhstan. When co-occurring with pneumonia, the risk of adverse outcomes is markedly elevated. These results support the integration of diabetes management into COVID-19 clinical pathways and public health planning. As Kazakhstan continues to face high rates of noncommunicable diseases, strengthening chronic disease surveillance and developing coordinated response strategies will be critical to reduce future pandemic mortality. Further studies with larger samples are warranted to validate these findings and inform national policy.

## Supplemental Information

10.7717/peerj.20413/supp-1 Supplemental Information 1 De-identified EMR dataset of adults hospitalized with laboratory-confirmed COVID-19 in Almaty, Kazakhstan (2021–2022), including comorbidities (diabetes/respiratory) and discharge outcomes (N=292). Each row corresponds to one adult patient hospitalized with laboratory-confirmed COVID-19 in 2021–2022 at a multidisciplinary hospital in Almaty, Kazakhstan (N=292). The dataset contains coded variables for age group, sex, nationality, marital status, diabetes (DM_code), pneumonia, chronic bronchial disease, and in-hospital vital status/discharge outcome; all personal identifiers have been removed. Data were extracted and analyzed in September–December 2023. The data support all bivariable and multivariable regression models presented in the article.

10.7717/peerj.20413/supp-2 Supplemental Information 2 Data Dictionary for the Almaty COVID-19 EMR dataset (2021–2022). This data dictionary defines every variable in the de-identified EMR dataset of 292 adult COVID-19 hospitalizations in Almaty (2021–2022), including variable name, label, definition, units, coding schemes (e.g., 0/1 or 1/2 with category labels), allowed values/ranges, and missing-value conventions. It also reports non-missing/missing counts, unique values, and example entries. The dictionary maps 1:1 to the single worksheet (“Sheet2”) and supports reproducibility of descriptive statistics, bivariate tests, and multivariable models reported in the manuscript.

10.7717/peerj.20413/supp-3 Supplemental Information 3 STROBE Checklist

## Additional Information and Declarations

### Data Availability

The following information was supplied regarding data availability: The anonymized raw dataset used for statistical analysis (variables on patient age, sex, comorbidities (diabetes, pneumonia, chronic bronchitis), and clinical outcomes (death, improvement, no change, mechanical ventilation)) is available in the Supplementary File.
