# Supplementary material for: Diabetes and respiratory diseases as comorbid conditions in COVID-19 patients from Kazakhstan: a retrospective pilot study
Source: PeerJ. 2025 Dec 16;13:e20413. doi: 10.7717/peerj.20413 (PMC12716145; doi:10.7717/peerj.20413)
Supplement: Supplemental Information 3 [file peerj-13-20413-s003.docx]

# STROBE Checklist – Completed for Submission

| Item | Recommendation | Page / Section |
| --- | --- | --- |
| 1 | Indicate the study’s design in title or abstract | Title and Abstract |
| 2 | Explain scientific background and rationale | Introduction, p.3 |
| 3 | State specific objectives | End of Introduction, p.5 |
| 4 | Present key elements of study design | Materials & Methods, Study Design, p.5 |
| 5 | Describe the setting, locations, and dates | Materials & Methods, Setting, p.5 |
| 6 | Eligibility criteria and methods of selection | Materials & Methods, Sampling and Participants, p.5–6 |
| 7 | Define all outcomes, exposures, confounders | Materials & Methods, Sampling and Participants, p.5 |
| 8 | Sources of data and measurement methods | Materials & Methods, Data Collection, p.6 |
| 9 | Address potential sources of bias | Discussion, Limitations, p.13 |
| 10 | Explain how the study size was determined | Materials & Methods, Sampling, p.5 |
| 11 | Handling of quantitative variables | Materials & Methods, Statistical Analysis, p.6 |
| 12 | Statistical methods | Materials & Methods, Statistical Analysis, p.6–7 |
| 13 | Participant numbers at each stage | Results, first paragraph, p.9 |
| 14 | Descriptive characteristics of participants | Results, Table 1 + p.7–8 |
| 15 | Outcome events or summary measures | Results, Table 2–3 + p.8–9 |
| 16 | Estimates and confidence intervals | Results, Table 2–3 + p.8–9 |
| 17 | Other analyses: subgroups, sensitivity | Results, Table 3 + p.9 |
| 18 | Summarize key results | Discussion, beginning, p.10 |
| 19 | Discuss study limitations | Discussion, Limitations, p.13 |
| 20 | Interpretation of results | Discussion, Interpretation, p.10–13 |
| 21 | Generalisability of results | Discussion, last paragraph, p.13 |
| 22 | Source of funding and role of funders | Acknowledgment, p.14 |
